# Separating the wheat from the chaff: mitigating the effects of noise in a plastome phylogenomic data set from *Pinus* L. (Pinaceae)

**DOI:** 10.1186/1471-2148-12-100

**Published:** 2012-06-25

**Authors:** Matthew Parks, Richard Cronn, Aaron Liston

**Affiliations:** 1 Department of Botany and Plant Pathology, Oregon State University, Corvallis, OR 97331-2902, USA; 2 Pacific Northwest Research Station, USDA Forest Service, Corvallis, OR 97331, USA

**Keywords:** Phylogenetic noise, Plastome, *Pinus*, Chloroplast

## Abstract

**Background:**

Through next-generation sequencing, the amount of sequence data potentially available for phylogenetic analyses has increased exponentially in recent years. Simultaneously, the risk of incorporating ‘noisy’ data with misleading phylogenetic signal has also increased, and may disproportionately influence the topology of weakly supported nodes and lineages featuring rapid radiations and/or elevated rates of evolution.

**Results:**

We investigated the influence of phylogenetic noise in large data sets by applying two fundamental strategies, variable site removal and long-branch exclusion, to the phylogenetic analysis of a full plastome alignment of 107 species of *Pinus* and six Pinaceae outgroups. While high overall phylogenetic resolution resulted from inclusion of all data, three historically recalcitrant nodes remained conflicted with previous analyses. Close investigation of these nodes revealed dramatically different responses to data removal. Whereas topological resolution and bootstrap support for two clades peaked with removal of highly variable sites, the third clade resolved most strongly when all sites were included. Similar trends were observed using long-branch exclusion, but patterns were neither as strong nor as clear. When compared to previous phylogenetic analyses of nuclear loci and morphological data, the most highly supported topologies seen in *Pinus* plastome analysis are congruent for the two clades gaining support from variable site removal and long-branch exclusion, but in conflict for the clade with highest support from the full data set.

**Conclusions:**

These results suggest that removal of misleading signal in phylogenomic datasets can result not only in increased resolution for poorly supported nodes, but may serve as a tool for identifying erroneous yet highly supported topologies. For *Pinus* chloroplast genomes, removal of variable sites appears to be more effective than long-branch exclusion for clarifying phylogenetic hypotheses.

## Background

The potential influence of phylogenetic ‘noise’, i.e. random or misleading signal, in molecular phylogenetic studies has been recognized for over 30 years [1-4]. Similarly, various strategies to identify and/or mitigate noise in datasets have been formulated, including measuring skewness in the distribution of phylogenetic trees [5,6], quantifying incongruence between data partitions [7], but see, for example, [8,9], likelihood mapping [10], increasing taxon sampling [11,12], and profiling loci based on phylogenetic information content [13-15], among others. While the specific details of these strategies differ, ultimately the goal of each is to increase the accuracy of phylogenetic hypothesis generation by identifying and/or reducing the influence of misleading signal [16]. Nonetheless, as next-generation technologies continue to bring about orders-of-magnitude increases in DNA sequence output and usher in an era of phylogenomics, the challenges associated with phylogenetic noise could temper gains in phylogenetic resolution resulting from increased taxon and sequence sampling [17-22]. Although genomic-scale data sets are relatively novel, it is clear that misleading signal inherent in these large datasets still impacts phylogenetic resolution, in particular in clades that have experienced rapid divergence or radiation events, as well as lineages with elevated rates of evolution and/or long periods of genetic isolation (i.e., “long branches”) [12,20,21,23-26].

While chloroplast sequences are still the most commonly used markers in plant phylogenetic studies, many analyses tend to rely on relatively small portions of the chloroplast genome, and few studies to date have applied plastome-scale sequences to phylogenetic questions [27-34]. This is particularly true at low taxonomic levels [31], as the majority of plastome-level phylogenetic analyses have focused on clarifying plastid-based relationships at familial and ordinal levels. Considering the potential impact of phylogenetic noise in phylogenomic analyses [17-22,35], it seems appropriate to explore such effects on plastome-scale datasets, particularly as they become widespread in plant phylogenetic analyses and more commonly applied to investigations at low taxonomic levels. Further, although representing a single linkage group, mutation rate varies between different regions of the plastome [31,36,37], and so the potential for misleading signal certainly exists when using full plastomes to delineate evolutionary events over varying time-scales.

**Figure 1:**
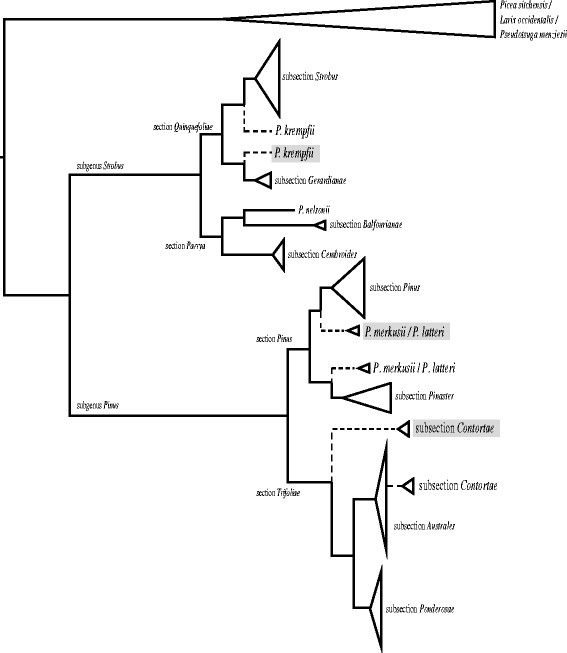
** Phylogenetic hypotheses for genus *Pinus* .** Alternate placements (indicated by dashed lines) of subsections *Contortae* and *Krempfianae*, as well as the clade consisting of *Pinus merkusii * and *P*. *latteri * are shown. The most common plastid-based resolution of these groups is indicated by gray shading. Tree topology and relative branch lengths reflective of data from Gernandt et al. [42], Parks et al. [31] and this study.

The genus *Pinus*, consists of ca. 110 species distributed primarily throughout the northern hemisphere, and contains evolutionary patterns ranging from deep divergence events to apparent rapid and relatively shallow radiations. In addition, the moderate size of the genus facilitates thorough taxon sampling. *Pinus * is represented by a relatively well-documented fossil record reaching back over 100 million years [38-40] and has been the focus of a large body of phylogenetic work, including studies based in morphology [41-45], crossability [41,46-48] and molecular data, including restriction fragment analyses [49,50] and both nuclear [51-54] and chloroplast sequence data [31,42,44,55-59]. The most recent molecular systematic treatment of *Pinus*[42] recovered a well-supported systematic framework consisting of two subgenera (*Pinus * and *Strobus *), four sections (sections *Pinus* and *Trifoliae* in subgenus *Pinus*, sections *Parrya* and *Quinquefoliae * in subgenus *Strobus *) and 11 subsections (Figure 1) that is widely accepted today. However, while nearly complete plastome sequences for a subset of pine species support this framework and result in increased resolution across much of the genus [31], there remain a number of taxa with poor resolution and/or incongruence between chloroplast-based and nuclear- or morphology-based analyses. In particular, subsections *Krempfianae * and *Contortae *, as well as a clade of the two closely related species *Pinus merkusii * and *P*. *latteri * each demonstrate these conflicts (Figure 1). In the present study, we investigated poor and conflicting resolutions in these clades using highly variable alignment positions and long-branches as proxies for phylogenetic noise. Sequential removal of variable sites and long branches was applied to the phylogenetic analysis of a full-plastome alignment which included most of the world’s pine species and several Pinaceae outgroups. While responses to these treatments differed between these three clades, each case provided insight into both the general patterns of response to noise removal in a phylogenomic dataset as well as specific characteristics of the plastid-based *Pinus * evolutionary history.

## Methods

### Accessions used in study

A total of 113 accessions were included in the alignment and subsequent analyses described below, including 37 *Pinus * and Pinaceae accessions utilized by Cronn et al. [60] and Parks et al. [61] (GenBank FJ899555-FJ899583, EU998739-998746, NC_001631.1 [62] and NC_004677.2) and the plastome sequence of *Cathaya argyrophylla * reported by Lin et al. [34] (GenBank AB547400.1) (Additional File 1). The 75 novel plastome accessions included in analyses were sequenced and assembled as follows:

### Genomic DNA extraction, chloroplast enrichment and sequencing

Total genomic DNA was extracted from fresh-frozen leaf or mega-gametophyte tissues using the FastDNA extraction protocol (MP Biomedicals, Ohio, USA). In several cases (*Pinus chiapensis *, *P*. *cembroides *, *Pinus dabeshanensis *, *P*. *discolor *, *P*. *douglasiana *, *P*. *edulis*, *P*. *hwangshanensis *, *P*. *massoniana *, *P*. *pumila *, and *P*. *sabiniana*), genomic DNA yield was insufficient for sequence preparation, so extracts were amplified by whole genome amplification with random hexamer priming [63]. Genomic libraries were prepared following the Illumina protocol [64], with fragmentation performed using a BioRuptor Sonicator (Diagenode, Inc., Denville, NJ, USA) (setting ‘high’ for 5–30 one minute cycles). Adapters ligated to genomic fragments carried unique 4 bp ‘barcodes’ at their 3´ ends for multiplex sequencing as described in Cronn et al. [60]. Agarose gel size-selected (300–700 bp), adapter-ligated libraries were enriched through 12–18 cycles of PCR using Phusion DNA polymerase and HF Buffer (New England Biolabs, Ipswich, MA, USA) and standard Illumina paired-end primers, and quantified using a Nanodrop 1000 (ThermoFisher Scientific, Wilmington, DE, USA).

Solution-based enrichment of the chloroplast portion of genomic libraries followed the general methods of Gnirke et al. [65]. Enrichments were performed as follows. Chloroplast probe pools were synthesized by first PCR-amplifying the entire plastome of a member of *Pinus * subgenus *Pinus * (*Pinus thunbergii *), using the methods described in Cronn et al. [60]. In addition, plastome regions unique to *Pinus* subgenus *Strobus * were amplified from *P*. *koraiensis * to account for regions not present in the *Pinus * subgenus *Pinus * plastome. PCR products were quantified using a Nanodrop 1000 and pooled in an equimolar mix. Pooled amplicons were blunted-ended and subsequently ligated into ‘concatemers’ (Quick Blunting Kit and Quick Ligation Kit, New England Biolabs, Ipswich, MA, USA) and purified with Agencourt AMPure beads (Beckman-Coulter Genomics, Danvers, MA, USA). Concatemer probe pools were denatured into single-stranded product using 0.4 N KOH, and then amplified and biotinylated in a single incubation of 18 h at 30°C in the presence of 5´-end biotinylated random hexamers, 0.4 mM biotin-14-dCTP stock, 1 mM dNTPs and φ29 DNA polymerase. After cleaning by ethanol precipitation, this procedure typically yielded pools consisting of 10-25 μg of large (tens of kbp in length) biotinylated chloroplast probe. Hybridization reactions were carried out in 40 μl volumes and contained 0.5 μg probe and 0.5–1 μg of either a single enriched genomic library or equimolar-pooled 4-plex genomic libraries; Denhardts solution (Invitrogen, Inc., Carlsbad, CA, USA) and lambda DNA (New England Biolabs, Ipswich, MA, USA) were used as blocking agents to minimize binding of non-target DNA to probes. Reactions were heated to 95°C for 10 min, and subsequently incubated at 65°C for 64–72 h. After incubation, hybridization products were captured using MagnaSphere streptavidin-coated paramagnetic beads (Promega, Inc., Madison, WI, USA). Capture reactions were incubated for 30 min at room temperature, after which the streptavidin-probe-target DNA complexes were captured through magnetization and then washed four times at 65°C in the presence of 0.1% SDS and 1X, 1X, 0.5X and 0.1X SSC for 15, 10, 10 and 10 min, respectively. Enriched hybrids were eluted from the paramagnetic beads in 50 μl dH2O at 80°C for 10 min and PCR-amplified over 12-18 cycles using Phusion-Flash PCR Master Mix (New England Biolabs, Ipswich, MA, USA) and standard Illumina paired end primers. After PCR enrichment, libraries were purified with Agencourt Ampure beads and subsequently quantified using the Nanodrop 1000, and size-confirmed using either gel electrophoresis or the Agilent 2100 BioAnalyzer (Agilent, Santa Clara, CA, USA).

The molarity of enriched libraries was estimated by their concentration and average fragment size, after which the libraries were submitted for sequencing singly or in barcode-specified multiplex pools ranging in size from four to 16 accessions. Individual samples or multiplex pools were submitted to the Oregon State University Center for Gene Research and Biocomputing (OSU CGRB) ( http://www.cgrb.oregonstate.edu/) or the FAS Center for Systems Biology at Harvard University ( http://sysbio.harvard.edu/csb/) for sequencing on Illumina GAIIx sequencers. Libraries were loaded at a concentration of 5–7 ρM and sequenced in 60 or 80 bp single-end sequencing reactions. Cluster formation, primer hybridization and sequencing reactions followed Illumina protocols [64]. Image analysis, base-calling and error estimation were performed using the Illumina GA Pipeline version 1.5.

### Plastome assembly from microreads

To initially determine enrichment of read pools, all reads containing Illumina adapter sequence were removed from read pools and the remaining reads were sorted by barcode using two Perl scripts, sort_fastq.pl and bcsort_fastq_se.pl (available at http://brianknaus.com). The proportion of reads representing the chloroplast was checked using the program BLAT [66] with default settings and a reference of either *Pinus thunbergii * or *P*. *koraiensis * for accessions in subgenus *Pinus * or *Strobus *, respectively.

Reference-guided assembly of microreads was facilitated using a pipeline of five scripts called "alignreads”, as described in Straub et al. [67]. In this pipeline, assembly of microreads into contigs is performed by YASRA [68], which assembles contiguous sequences (contigs) by iteratively aligning sequence reads to a reference genome using the lastz alignment algorithm [69]. The alignment of assembled contigs is then refined using NUCmer and Delta-Filter of the MUMmer 3.0 suite [70], and the resulting alignment information is paired with the original contigs and read depth information from YASRA, to be converted into an aligned consensus sequence using sumqual.py and qualtofa.py. The latter allows user-specified masking of contig positions based on read depth and base call proportion. Both sumqual.py and qualtofa.py are available for download at http://milkweedgenome.org; YASRA and MUMmer are available online at http://www.bx.psu.edu/miller_lab/ and http://mummer.sourceforge.net/, respectively.

For assembly of the novel plastome sequences reported in this paper, subsectional references reported in Parks et al. [61] were used (Additional File 1). The alignment of assembled contigs was checked and adjusted manually in BioEdit 7.0.9 [71]. Aligned contig positions matching the reference were masked if fewer than five overlapping reads and less than 80% of all reads overlapping to form the contig at that position agreed with the reference; aligned positions called as SNPs were similarly masked, but required a minimum coverage depth of 20 aligned reads and 80% call proportion.

### Alignment and quality screening of assemblies

Plastome assemblies were aligned in MAFFT v.6.240 [72], using gap opening and extension penalties of 2.0 and 0.1, respectively. Alignments were subsequently manually adjusted and annotated in BioEdit 7.0.9 where necessary. In particular, MAFFT appeared to have the most difficulty with insertions and deletions, ranging from inconsistent arrangement of variable length homopolymer repeats in noncoding regions to clear failure in alignment of larger repetitive elements with variable copy number, for example in the loci *ycf *1 and *ycf *2. As our taxon sampling was relatively deep, in most cases it was possible to rely on the assumed homology of closely related species or groups of species to guide manual adjustment and, as much as possible, the direction toward a most parsimonious solution was favored. The plastome of *Cathaya argyrophylla * was primarily aligned by hand due to structural rearrangements. The assemblies of exonic regions were checked and adjusted as necessary by translation to identify potential misassemblies, as represented by internal stop codons and/or frameshift mutations.

Novel plastome sequences were quality-screened at this point by level of completion and relative similarity to the subsectional reference used in their assembly. Specifically, assemblies were discarded from further analyses if they were estimated to be less than 80% complete, or if the pairwise distance to their subsectional assembly reference was greater than two times the standard deviation of all pairwise distances between assembled members of their subsection and the subsectional reference. The latter measure was taken to diminish bias resulting from poor assemblies, for example resulting from low coverage or capture of divergent paralogous copies of chloroplast regions residing in the nuclear or mitochondrial genome. In addition, several assemblies were discarded due to poor overall assembly quality as evidenced by highly divergent exon/protein sequences and divergence from Sanger-sequenced plastome regions of the same species. Previously published *Pinus* plastome sequences were used only if they exceeded 80% estimated sequence completion.

### Phylogenetic analysis of full plastome alignment

Maximum likelihood (ML) and Bayesian phylogenetic analyses of the complete alignment (including the ca. 450 bp remnant of the IR common to members of Pinaceae) were completed through the Cipres Science Gateway ( http://www.phylo.org/) using RAxML-HPC2 [73] and MrBayes [74], both on the available teragrid. Maximum likelihood analysis in this case was performed under the GTRGAMMA model, with the number of bootstrap replicates automatically determined under the recommended autoMRE option, and gaps treated as missing data. Bayesian analyses were performed under the same model of evolution and with the same treatment of gapped positions. Each analysis consisted of two runs with four chains each (three hot and one cold chain), run for 10,000,000 generations with trees sampled every 1000 generations, and the first 25% of trees discarded as burn-in. Stationarity was evaluated by graphing –lnL of trees across all generations and by requiring the standard deviation of the two runs to be less than 0.05. All trees were combined from both runs past the point of stationarity to determine topology and support through the majority rule consensus tree using PAUP* v.4.0b10 [75]. Parsimony analysis was performed with PAUP* v.4.0b10, under heuristic search with ten repetitions of random sequence addition, tree bisection and reconnection branch swapping and 100 bootstrap replicates; gapped positions were again treated as missing data.

### Evaluation of the impact of variable site removal

Variable sites in the full plastome alignment were ranked using both tree-independent and tree-dependent methods – i.e. without and with the consideration of an underlying phylogenetic framework, respectively. In the tree-independent strategy, all alignment sites in the full plastome matrix were ranked based on their variability using the script sorter.pl [35], which measures the ‘observed variability’ (OV) of each position in an alignment as:

(1) $$\text{OV}=\text{sum}\left(1\ldots \text{k}\right)\left\{{\text{d}}_{\text{ij}}\right\}/\text{k}$$

where:

 k, the number of all possible pairwise comparisons between accessions in an alignment, excluding accessions with a gap or masked base at the position considered; dij, the score of character variability (0 for match, 1 for mismatch) in each of k pairwise comparisons of accessions in the alignment (accessions with gaps and masked positions excluded, as noted for k above).

Starting with the highest variability sites, alignment positions were serially removed from the full alignment in 100 site partitions using the script sorter.pl, resulting in two series of data partitions. Following Goremykin et al. [35], the first series, A_n_, consisted of all alignment positions except the most variable 100, 200, 300,…,20000 sites, while the second series, B_n_, consisted of the most variable 100, 200, 300,…,20000 sites. Our notation differs slightly from Goremykin et al. in that we use the subscript for A_n_ to refer to the size of a given A_n_ partition, while the subscript of B_n_ refers also to the size of the corresponding A_n_ partition.

In the tree-dependent strategy, the variability of all sites in the full plastome matrix was measured using the program AIR-Identifier [76] as implemented in the University of Oslo Bioportal ( https://www.bioportal.uio.no/). AIR-Identifier utilizes the baseml application of PAML [77,78] to estimate site variability in a nucleotide alignment within a maximum likelihood framework. For these analyses, the maximum likelihood phylogenetic tree from analysis of the full alignment (see above) was used as the underlying phylogenetic framework. Variability of alignment sites was quantified under default settings as recommended by the site’s author (S. Kumar, pers. comm.) (Additional File 2) and after several difficulties due to the size of our data matrix, and corresponding series of A_n_ and B_n_ matrices were constructed as described above up to the most variable 20000 sites, with the exception that variable sites were extracted from the alignment using PAUP* v.4.0b10.

For each strategy, phylogenetic analyses on all A_n_ and B_n_ data partitions were run through the OSU CGRB GENOME Cloud computing resources ( http://bioinfo.cgrb.oregonstate.edu) using RAxML-VI-HPC v.2.2.3 [73] primarily under the GTRGAMMA model (some of the larger partitions were run under the GTRCAT model due to time constraints), with 100 bootstrap replicates and gaps treated as missing data. The highest likelihood tree from each A_n_ partition and its corresponding B_n_ partition were then compared using the branch score metric [79] and partition metric [80] as implemented in the treedist executable of Phylip v.3.69 [81]. These comparisons allowed visual inspection of the effects of increasing variable site removal from the alignment. It was expected that the topology of corresponding A_n_ and B_n_ phylogenetic trees should converge as more sites are removed from A_n_ partitions and added to the B_n_ partitions, since variable sites partitions should increasingly contain accurate rather than misleading phylogenetic signal as the process of variable site removal progresses [35,82]. In turn, median bootstrap values and the distribution of bootstrap values were also recorded for all A_n_ partitions to visually assess the point at which excess phylogenetic signal was being lost through variable site removal (as evidenced by a decrease in overall bootstrap support values). Trends in bootstrap support values were then visually compared to trends in branch score and partition metrics to identify whether a ‘window’ representing the highest signal to noise ratio in a range of A_n_ partitions existed. If present, such a window would be found in A_n_ partitions corresponding to low topological differences between phylogenetic trees generated from A_n_ and corresponding B_n_ partitions, yet high overall bootstrap support in trees generated from A_n_ partitions.

Trends in topology and bootstrap support were subsequently investigated over the process of variable site removal specifically for three taxa with historically recalcitrant phylogenetic positions: 1) the monotypic subsection *Krempfianae*, consisting of the morphologically distinctive flat-needled *P*. *krempfii *, 2) the southeast Asian clade consisting of *P*. *merkusii * and *P*. *latteri *, and 3) subsection *Contortae *, consisting of *Pinus contorta *, *P*. *banksiana*, *P*. *clausa * and *P*. *virginiana *, (Figure 1). For these analyses, bootstrap values for the nodes immediately ancestral to all three taxa were recorded for each A_n_ partition, as these nodes represented the resolution between disputed alternative placements of each taxon (Figure 1). In addition, bootstrap values supporting the monophyly of the *P*. *merkusii */*P*. *latteri * clade and subsection *Contortae * were recorded for each A_n_ partition.

### Evaluation of the impact of long-branch exclusion

As a general rule, the *Pinus* chloroplast phylogeny contains relatively long branches (substantial divergence) separating the two subgenera and four sections, but relatively short branches (low divergence) within subsections [31,42]. As a result, to remove long branches it is necessary in most cases to remove entire clades at the subsectional level or higher. Because of this and due to the conflicting topologies of interest residing at the subsectional level, long branches were excluded in the following manners: 1) all six Pinaceae outgroups were removed prior to phylogenetic analyses, 2) only the subgenus of interest was included in the analyses, and 3) only the section of interest and one member of the sister section were included in analyses. For the most exclusive strategy, *P*. *monophylla * (EU998745.4), *P*. *ponderosa * (FJ899555.2) and *Pinus thunbergii* (NC_001631.1) were used as outgroups for sections *Quinquefoliae *, *Pinus * and *Trifoliae * respectively. Maximum likelihood phylogenetic analyses were performed as described above for the full alignment for each strategy of long-branch exclusion on each of three partition sizes of interest (full alignment, A_136665_, A_133065_, as discussed in Results).

### Impact of noise-removal strategies on saturation

To gain further insight into the impact of variable site removal and long-branch exclusion on saturation in our data matrix (i.e., the history of multiple nucleotide state changes at individual sites), pairwise genetic distances between all accessions were determined in MEGA4 [83] both without correction and with application of a Jukes-Cantor correction. The correlation of these values was determined by linear regression for each of three partition sizes of interest in the OV-based variable site removal analysis (full alignment, A_136665_, A_133065_, as discussed in Results) and for each strategy of long-branch exclusion. The slope of the regression line was taken as indicative of the level of saturation present in the dataset, such that higher values for corrected pairwise distances relative to uncorrected distances correspond to higher levels of saturation [18,84,85].

## Results

### Sequence assembly and alignment

After quality/chastity filtering through the Illumina GA Pipeline v. 1.5 and removal of adapter sequences, read pools for successfully assembled plastome sequences averaged 1.77 ± 0.76 million reads per accession, while chloroplast reads accounted for 56.83 ± 13.85% of these reads on average (SRA047299.1, Additional File 1). Seventy-five novel assembled plastome sequences averaged 117157 ± 3634 bp in length, and were estimated to be 98.1 ± 2.5% complete on average after masking (GenBank JN854151-JN854220, JN854222-JN854226, Additional File 1). The alignment of all successfully assembled plastome sequences, including 107 *Pinus* accessions and six Pinaceae outgroups, resulted in 141265 aligned sites. Within this alignment, 1217 positions were composed entirely of masked bases or gaps and masked bases due to failure of called nucleotides to meet coverage requirements. The complete alignment is available through TreeBASE ( http://www.treebase.org, study number 12640).

### Variable sites

**Figure 2:**
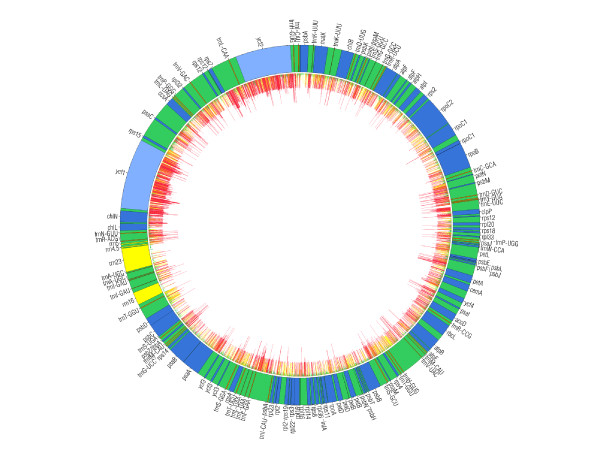
**Distribution of OV for variable plastome alignment positions.** Schematic of the *Pinus* chloroplast genome with annotated protein-coding exons (blue), rRNA loci (yellow), tRNA loci (orange) and noncoding regions (green). The coding loci *ycf*1 and *ycf*2 are highlighted in light blue. The distribution of OV values > 0 is indicated by the internal histogram, as follows: red – most variable 4.6 kbp (A_142165_ to A_136665_ ); yellow – most variable sites from 4.6 to 8.3 kbp (A_136565_ to A_133065_ ); green – remaining sites with OV > 0.

Variable sites were identified in nearly all coding and noncoding regions of the plastome, although they were unequally distributed between and among exons, introns and noncoding regions (Table 1, Figure 2). Highest average per-site OV was found in noncoding regions, followed by protein-coding exons, introns, and finally RNA-coding exons (Table 1). The higher variability of exons than introns was an unexpected result; however, previous work [31,61] has shown that the loci *ycf *1 and *ycf*2 are extremely variable in *Pinus * compared to other protein-coding loci. Because of this, OV calculations were also averaged for exons without *ycf*1 and *ycf*2. With the removal of either *ycf *1 alone or both *ycf*1 and *ycf*2 positions, average per site OV for protein-coding exons fell below that of intronic regions (Table 1), although the difference between intronic regions and exonic regions with removal of only *ycf*1 was not significant. The distribution of rate values for alignment positions by AIR-Identifier was similar, although intron regions were significantly more highly variable than all three exon partitions, and variability of tRNA loci was significantly higher on average than rRNA and exon regions with the exclusion of *ycf *1 and *ycf *2 (Additional File 3).

**Table 1:** Average per site OV values of plastome regions for full plastome alignment

	**Noncoding regions**	**Protein-coding exons**	**Introns**	**tRNA**	**rRNA**
average OV	0.04546^a^ (0.12833)	0.03153^b^ (0.11227)	0.02110^c^ (0.08880)	0.00443^e^ (0.03725)	0.00462^e^ (0.04255)
average OV without *ycf*1		0.01907^c^ (0.08184)			
without *ycf*1 or *ycf*2		0.01478^d^ (0.06997)			

Values given are for alignment of all 113 *Pinus* and Pinaceae accessions. Standard deviations are given in parentheses. Mean values with different superscript letters are significantly different at α < 0.05 in Tukey’s HSD test, following one-way ANOVA supporting different means at p < 0.0001.

### Phylogenetic analysis of the full alignment

Our full alignment contained 42468 alignment patterns, and resulted in highly supported and almost completely congruent topologies in ML, Bayesian and parsimony analyses (Additional File 4). All major clades at the subgenus, sectional and subsectional levels as reported by Gernandt et al. [42] were recovered with 95–100% bootstrap support. Across the topology, average ML bootstrap support for 105 ingroup nodes was 89.7% (standard deviation = 18.5%). Only two minor topological conflicts were found between methods. In subsection *Australes Pinus caribaea * was placed sister to a clade of *P*. *cubensis * and *P*. *occidentalis * with low support in Bayesian analysis (<0.6 posterior probability), while ML and parsimony analyses recovered *P*. *caribaea * sister to *P*. *palustris *, again with low support (≤50% bootstrap support). In section *Quinquefoliae *, parsimony analysis recovered *P*. *morrisonicola * in a weakly supported clade with *P*. *armandii* (55% bootstrap support), while both Bayesian and ML methods recovered these species in a grade with variable support (43% bootstrap support, 0.97 posterior probability). For the three clades of interest, topology was consistent between methods, while support was in some cases variable. For example, section *Quinquefoliae* was recovered as subsection *Strobus * + (*P*. *krempfii * + subsection *Gerardianae *) with weak to strong support (58–73% bootstrap / 1.0 posterior probability) for the position of *P*. *krempfii *. Section *Pinus * was recovered as subsection *Pinus * + (*P*. *merkusii */*P*. *latteri * + subsection *Pinaster *) with weak to moderate support for the position of *P*. *merkusii */*P*. *latteri * (50–71% bootstrap / 0.52 posterior probability) but strong support for the monophyly of these two species (100% bootstrap, 1.0 posterior probability).Section *Trifoliae* was recovered as subsection *Contortae* + (subsection *Australes* + subsection *Ponderosae*) with high support (100% bootstrap / 1.0 posterior probability) for the monophyly and position of subsection *Contortae *.

### Impact of variable site removal

#### Tree-independent strategy

**Figure 3:**
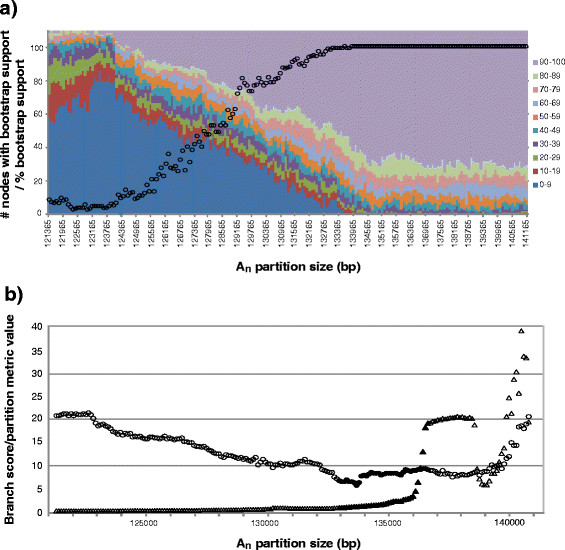
**Trends in bootstrap support values and topologies for likelihood analyses of alignment partitions.** For OV-based analyses, the following are shown: **a**) Distributions of bootstrap support values for all nodes. Circles represent median bootstrap support for each A_n_ partition size. **b**) Distribution of branch score metric (triangles) and partition metric (circles) values for tests of topological congruence between A_n_ and corresponding B_n_ data partitions. Filled data points correspond to A_n_ partitions sizes falling between final decrease of branch score metric values and start of decreases in overall bootstrap support values for A_n_ partitions. Partition metric values shown are 0.1× actual value in order to fit on same scale with branch score metric values.

Bootstrap support values showed clear trends throughout A_n_ partitions as variable sites were removed, with overall values consistently high (average value > 85%, median value ≥ 98%) until the most variable ca. 8.3 kbp had been removed (A_133065_) (Figure 3). Support steadily decreased from this point before leveling off at low values with removal of ≥18kbp of variable sites (mean/median bootstrap support <17% /<10%). Branch score metric values decreased relatively rapidly over removal of the first ca. 2kbp of highly variable alignment positions (A_141265_ to A_139265_), but subsequently rose again and plateaued at about half their maximal level from ca. A_138565_ to A_136665_, until decreasing rapidly again and remaining at low levels (Figure 3). Partition metric values showed an initial rapid decline before leveling off after the removal of the most variable 2.2 kbp (ca. A_139165_) (Figure 3), at which point values remained constant and relatively low until increasing again beyond removal of the most variable 8.3 kbp (A_133065_). The most highly variable 2kbp of alignment positions, corresponding to the first rapid decline in branch score and partition metric values, was dominated by positions wherein the majority of taxa contained gaps and masked bases (mean/median/standard deviation of gaps + masked bases per alignment position = 80.49/108/44.05). Visual inspection of A_n_ and B_n_ trees further revealed that the great majority of topological differences over the plateau of branch score metric values from ca. A_138565_ to A_136665_ involved changes in branch lengths associated with subgeneric and sectional level divisions. This is also indirectly evidenced by the consistently low values of partition metric scores over this range, which reflect the consistent branching orders between A_n_ and B_n_ partitions but do not reflect differences in branch lengths. It is likely that these branch length differences are largely responsible for the temporary increase in branch score metric values seen here. Such a result is not completely unexpected, as variable sites associated with internal divisions of large groups of taxa typically have relatively high OV scores [35].

**Figure 4:**
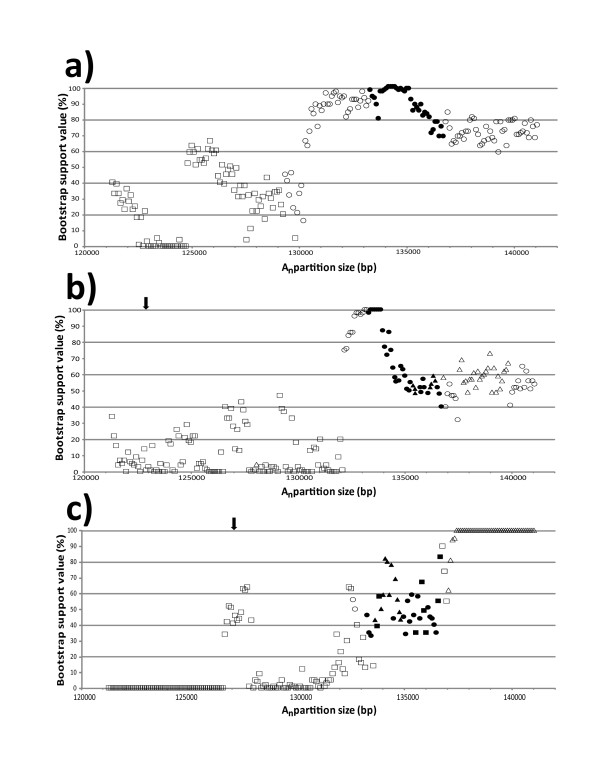
**Distribution of bootstrap support values for phylogenetic position of three clades in genus *Pinus* **. **a**) Bootstrap support values for placement of subsection *Krempfianae *. Circles correspond to placement of *P*. *krempfii * sister to subsection *Gerardianae *. **b**) Bootstrap support values for placement of *Pinus merkusii * / *P*. *latteri *. Circles correspond to placement of *P*. *merkusii*/*P*. *latteri* as sister to subsection *Pinaster* and triangles as sister to subsection *Pinus*. **c**) Bootstrap support values for placement of subsection *Contortae*. Circles correspond to placement of subsection *Contortae* as sister to subsection *Australes * and triangles as basal to both subsections *Australes* and *Contortae*. For all charts, filled data points correspond to A_n_ partition sizes falling between final decrease of branch score metric values and start of decrease in overall bootstrap support values for A_n_ partitions, as shown in Figure 3. Squares represent variable phylogenetic placements not including those represented by circles or triangles. Arrows in b) and c) indicate partition size at which bootstrap support for monophyly of clade falls below 100%.

Bootstrap support for the phylogenetic position of *P*. *krempfii* was moderate (59–84%) until removal of the most variable 5.7 kbp (ca. A_135665_), at which point bootstrap values steadily increased until peaking at 97–100% after removal of the most variable 6.3–7.8 kbp (ca. A_135065_ to A_133665_) (Figure 4). A_n_ partitions greater than 129.4 kbp in size recovered section *Quinquefoliae * as subsection *Strobus * + (*P*. *krempfii * + subsection *Gerardianae*); at A_n_ partition sizes smaller than this phylogenetic position was variable.

The monophyly of *P*. *merkusii*/*P*. *latteri* was highly supported until removal of the most variable 18.2 kbp (A_123165_) (Figure 4). Support for their resolution within section *Pinus*, however, was consistently moderate until removal of 7.2 kbp of the most variable sites (A_134165_). A_n_ partitions prior to this point recovered the *P*. *merkusii*/*P*. *latteri* clade alternately sister to subsection *Pinaster* and subsection *Pinus*. After this point, bootstrap support rapidly increased to a peak of 96–100% between removals of 7.6–9 kbp of the most variable sites (ca. A_133765_ to A_132465_), and all A_n_ partitions in this range recovered section *Pinus* as subsection *Pinus* + (*P*. *merkusii*/*P*. *latteri* + subsection *Pinaster*).

Monophyly of subsection *Contortae* was highly supported until removal of 15.3 kbp of the most variable sites (ca. A_126065_), while support for the phylogenetic position of the *Contortae* decreased fairly steadily after removal of only 4.2 kbp (ca. A_137265_) (Figure 4). Section *Trifoliae* was recovered as subsection *Contortae* + (subsection *Australes* + subsection *Ponderosae*) by all A_n_ partitions greater than 137 kbp in size; resolution based on A_n_ partitions less than 137 kbp in size was variable, although placement of subsection *Contortae* as sister to or nested within subsection *Australes* was supported by several partitions between A_136665_ and A_133065_.

#### Tree-dependent strategy

Overall, trends were not as clear using the tree-based approach employed. In particular, bootstrap support values remained constant and high, with a median value of 100% in all A_n_ partitions (Additional File 5). A_n_ and B_n_ tree topological comparisons showed somewhat similar trends as seen in the OV-based strategy, with rapid initial decreases followed by consistently low values in branch score and partition metric values (Additional File 5); however due to the lack of change in bootstrap support values, it was not possible to identify a target window of variable site removal using this method.

Nonetheless, phylogenetic patterns in the three clades of interest were partly reflective of tree-independent results, suggesting an influence of highly variable sites in the placement of at least two of these clades. For example, *P*. *krempfii* was placed as sister to subsection *Gerardianae* by all A_n_ partitions, and reached high support with variable site removal. In this case removal of both 8.8-8.9 kbp and 14.7-20 kbp resulted in 100% bootstrap support (Additional File 5). *P*. *merkusii*/*P*. *latteri* also showed increased support with removal of variable sites and, similar to tree-independent results initially varied in phylogenetic placement as sister to subsection *Pinus* and subsection *Pinaster* (Additional File 6). Support for (subsection *Pinus* + *P*. *merkusii*/*P*. *latteri*) peaked at 97% with removal of 9.7–10.5 kbp, while support for (subsection *Pinaster* + *P*. *merkusii*/*P*. *latteri*) reached 100% with removal of 15.3–20 kbp. In contrast to tree-independent results, subsection *Contortae* was found as sister to (subsection *Australes* + subsection *Ponderosae *) with 100% bootstrap support for all A_n_ partitions (Additional File 6).

### Impact of long-branch exclusion

When all alignment sites were included in analyses, long-branch exclusion strategies had essentially no impact on the topology or support of subsection *Krempfianae * or *Contortae *, while support increased moderately for a monophyletic (*Pinus merkusii*/*P*. *latteri * + subsection *Pinaster *) only with the most exclusive strategy (Table 2). When long-branch exclusion was used in combination with variable site removal (specifically partitions A_136665_ and A_133065_), trends were reflective of variable site removal alone for partition size A_136665_ in subsection *Contortae * and *P*. *merkusii */*P*. *latteri* (Table 2). In the remaining cases, trends were either non-existent (*P*. *krempfii* and *P*. *merkusii */*P*. *latteri * exclusion strategies applied to A_133065_) or counter to patterns seen with variable site removal alone (*P*. *krempfii * exclusion strategies applied to A_136665_, subsection *Contortae* exclusion strategies applied to A_133065_) (Table 2).

**Table 2:** **Impact of long-branch exclusion on full alignment, A**_**136665 **_**and A**_**133065 **_**data partitions**

	**Subsection *Krempfianae* ( *P* **. ** *krempfii* )**				** *Pinus merkusii* / *P* **. ** *latteri* **				**Subsection *Contortae* **			
	**All**	**No non- *Pinus* outgroups**	**Subgenus only**	**Section + single outgroup**	**All**	**No non- *Pinus* outgroups**	**Subgenus only**	**Section + single outgroup**	**All**	**No non- *Pinus* outgroups**	**Subgenus only**	**Section + single outgroup**
Full Alignment	(K + G) + S	(K + G) + S	(K + G) + S	(K + G) + S	(M/L + Pina.) + Pinus	(M/L + Pina.) + Pinus	(M/L + Pina.) + Pinus	(M/L + Pina.) + Pinus	C + (A + P)	C + (A + P)	C + (A + P)	C + (A + P)
	(79)	(61)	(79)	(79)	(53)	(53)	(54)	(78)	(100)	(100)	(100)	(100)
A_136665_	(K + G) + S	(K + G) + S	(K + G) + S	(K + G) + S	(M/L + Pina.) + Pinus	(M/L + Pina.) + Pinus	(M/L + Pina.) + Pinus	(M/L + Pina.) + Pinus	C + (A + P)	C + (A + P)	C + (A + P)	C + (A + P)
	(71)	(70)	(71)	(54)	(43)	(46)	(47)	(68)	(100)	(100)	(100)	(100)
A_133065_	(K + G) + S	(K + G) + S	(K + G) + S	(K + G) + S	(M/L + Pina.) + Pinus	(M/L + Pina.) + Pinus	(M/L + Pina.) + Pinus	(M/L + Pina.) + Pinus	P + (C + A)	P + (C + A)	P + (C + A)	C + (A + P)
	(97)	(97)	(97)	(98)	(99)	(100)	(100)	(99)	(37/42)	(31/36)	(35/42)	(84)

For each combination, supported topology is given with maximum likelihood bootstrap support underneath in parentheses. Subsection and species abbreviations are as follows: A = *Australes *, C = *Contortae *, G = *Gerardianae *, K = *P*. *krempfii *, M/L = *P*. *merkusii */*P*. *latteri *, P = *Ponderosae*, Pinast. = *Pinaster * , S = *Strobus *. Single outgroups used in most exclusive groups are described Methods and Materials.

### Impact of variable site and long-branch removal on saturation

Based on the slopes of regression lines of corrected vs. uncorrected pairwise distances, saturation decreased similarly both with OV-based variable site removal and long-branch exclusion strategies (Table 3, Additional File 7). The highest levels of saturation were observed with inclusion of all accessions, while the lowest values occurred with removal of the most variable 8.3 kbp of the alignment (A_133065_) and exclusion of at least the Pinaceae outgroups (Table 3).

**Table 3:** Slopes of regression lines for plots of corrected versus uncorrected pairwise distances

	**All accessions**	**No non- *Pinus* outgroups**	**Subgenus *Pinus* **	**Subgenus *Strobus* **	**section Q *uinquefoliae* ** **+** ** *P. monophylla * **	**section *Pinus * ** **+** ** *P. ponderosa* **	**section *Trifoliae* ** **+** ** *P. thunbergii * **
Full alignment	0.9574	0.9682	0.9898	0.9893	0.9905	0.9925	0.9928
	(0.9571-0.9577)	(0.9681-0.9683)	(0.9895-0.9898)	(0.9888-0.9898)	(0.9893-0.9918)	(0.9911-0.9939)	(0.9921-0.9935)
A_136665_	0.9604	0.9851	0.9935	0.9903	0.9916	0.9941	0.9948
	(0.9601-0.9607)	(0.9850-0.9853)	(0.9929-0.9941)	(0.9897-0.9908)	(0.9903-0.9928)	(0.9923-0.9958)	(0.9939-0.9958)
A_133065_	0.9655	0.9954	0.9959	0.9950	0.9952	0.9955	0.9961
	(0.9653-0.9656)	(0.9950-0.9958)	(0.9953-0.9964)	(0.9938-0.9962)	(0.9932-0.9972)	(0.9935-0.9976)	(0.9953-0.9969)

95% confidence intervals for slopes are shown in parentheses. Slopes with values below 1.0 represent increased levels of saturation in the alignment tested. Intercepts of all lines were significantly different than zero at p < ≤0, with the exception of section *Pinus* + *P*. *ponderosa* for treatment A_133065_, which was not significantly different than zero. 95% confidence intervals for intercepts significantly different than zero were all 1.8 × 10^-6^ ≤ y ≤ 0.00040.

## Discussion

As genome-scale datasets become increasingly common in evolutionary analyses, it is reasonable to expect challenges associated with highly variable or noisy data. Because of this, it is prudent to develop efficient strategies to identify and mitigate phylogenetic noise while simultaneously preserving sites and taxa carrying useful phylogenetic signal in order to most effectively capture information from large datasets. The benefit of developing such strategies has been demonstrated already, for example in placental mammals [35], early-diverging plant lineages [82,86] and deep eukaryotic phylogeny [84]. Our approach is similar to previous efforts, but focused on two fundamental and complementary strategies, variable site removal and long-branch exclusion, and explored the dynamics of tree topology and support values to measure their impact on an infrageneric phylogenetic analysis. While the two strategies employed were both utilized to counter the effect of putative phylogenetic noise, there are important contrasts between them. For example, the strict application of long-branch exclusion serves to minimize long-branch attraction artefacts, yet phylogenetic hypotheses may still be misled by evolutionary patterns at highly variable sites since all sites are still included in the analysis. In this case, removal of taxa could mask evolutionary patterns at some sites that otherwise might be more clearly interpreted [4,87], while the inclusion of fast-evolving sites may still mislead phylogenetic analyses [13]. On the other hand, removal of highly variable sites potentially diminishes the impact of misleading signal in an alignment and should increase the ability of applied models of sequence evolution to capture evolutionary patterns in phylogenetic analyses. The success of this strategy may be limited, however, as the inclusion of highly divergent taxa could still lead to long-branch artefacts when phylogenetic signal is minimal, and the broad application of variable site removal may have the unintended result of diminishing or erasing phylogenetic signal in some sub-clades [88]. It is therefore likely that utilizing a combination of these two strategies is prudent in many cases [84], yet an overly conservative approach could still lead to the loss of essential phylogenetic signal.

With our dataset and strategies, removal of variable sites in a tree-independent manner appears to provide more information toward clarifying the plastid-based evolutionary relationships of three historically problematic clades than does the exclusion of long branches. Further, removal of up to several thousand of the most variable alignment positions as measured by the OV metric was useful in investigating conflicting or weakly supported phylogenetic resolution in these clades; however, it is important to note that this magnitude of variable site removal is specific to *Pinus* and is expected to vary in other taxa depending on their evolutionary histories and the amount and origin of the sequence utilized in phylogenetic analyses. Nonetheless, the targeted range of variable site removal in this study (from ca. 4.3 to 8.6 kbp of highly variable sites), corresponding to alignment partitions with lower branch score/partition metric values and high overall bootstrap support, is significant in two regards. First, as overall high levels of bootstrap support are maintained across this range of partitions (Figure 3), sites within this window are not required for the resolution of most intraspecific relationships within the genus *Pinus*. Second, as variable sites are removed within this range of partitions important changes are seen in the topology and support of three historically recalcitrant clades - support for the position of subsection *Contortae* as sister to the monophyletic grouping of subsections *Australes* and *Ponderosae* diminishes substantially (discussed below), while there is substantial increase in resolution for the consistent positions of *P*. *krempfii* and *P*. *merkusii* / *P*. *latteri*. Conversely, the long-branch exclusion strategies applied have little to no effect on the topology and support for these clades when applied to the full plastome alignment, suggesting that variable site removal is more effective in mitigating what we perceive to be the impact of phylogenetic noise in our data set.

Finally, the tree-dependent strategy applied to our dataset also did not have the same impact as the OV-based strategy in relation to the three clades of interest. Rather, it appeared that this procedure was more strongly influenced by positions primarily delimiting generic and subgeneric divisions, as evidenced in part by the relatively high variability categorization of rRNA positions (Additional File 3). Critically, as overall bootstrap support values did not change even with extensive variable site removal, it was not possible to identify a finite window of putative high signal to noise ratio as with the tree-independent strategy. It is possible that this method would be more efficient if combined with the exclusion of outgroups or if applied to clades consisting solely of more closely related taxa. Alternatively, it may be that tree-based methods are in many cases limited by the influence of the assumed phylogenetic hypothesis [89]. The absence of change in overall bootstrap support values with tree-dependent variable site removal in our dataset supports the latter contention.

The specific changes in position and topological support shown in our analyses are also noteworthy because they highlight the disparate resolutions previously supported by different analyses or different types of data. For example, the unique morphological characteristics of *Pinus krempfii* (most notably its flat, paired needles) have led to a wide range of phylogenetic resolution, including placement outside the genus *Pinus*[90], in its own subgenus within *Pinus*[41,91,92], and within subgenus *Strobus *, section *Parrya*[93-95]. At least two recent morphological treatments have recognized some affinity of *P*. *krempfii * to *P*. *gerardiana * and *P*. *bungeana * of subsection *Gerardianae*, based on cuticular micromorphology and the morphology of tracheid and parenchyma cells [94,96]. Molecular evidence to date strongly support a position within or sister to section *Quinquefoliae * of subgenus *Strobus*, although a consistent and clear relationship of *P*. *krempfii * to subsections *Strobus* and *Gerardianae* of section *Quinquefoliae* has proven elusive (Figure 1). Some analyses based on chloroplast sequence data suggest an affinity to subsection *Gerardianae*[56,57], but support for this relationship is typically moderate to weak. Other reports based on chloroplast or nuclear sequence data show poor resolution [42,44], place the species sister to section *Quinquefoliae *[31], or suggest inclusion within subsection *Strobus *[51,52].

*Pinus merkusii* and *P*. *latteri * have demonstrated similarly ambiguous phylogenetic resolution relative to subsections *Pinus* and *Pinaster * of section *Pinus* (Figure 1), and again there is incongruence between molecular and morphological data. For example, Frankis [45] placed *P*. *merkusii * within subsection *Pinaster * based on cone morphology, while most molecular analyses place *P*. *merkusii * as sister to subsection *Pinus *[42,51,52,57], albeit typically with low to moderate support. On the other hand, Wang et al. [56,57] and Szmidt et al. [97] demonstrated a clear genetic separation of *P*. *merkusii * from sampled Asian members of subsection *Pinus*, and suggest a divergence between these groups possibly in the early Tertiary, although this timeframe is not in accordance with the age of section *Pinus* based on molecular clock calibrations [40,98].

Finally, the position of subsection *Contortae* is strongly supported (up to 100% bootstrap support) as sister to subsections *Ponderosae * and *Trifoliae * (Figure 1) based on previous reports using chloroplast sequence data or chloroplast restriction fragment analyses [31,42,44,49,55,59]. Alternatively, other lines of evidence suggest this highly supported topology may be incorrect. For example, hybridization is possible between some members of subsections *Contortae* and *Australes*, but not between members of subsections *Contortae* and *Ponderosae*[99,100]. Similarly, the relatively shallow fossil record of subsection *Contortae*[101,102] suggests a more recent derivation within its section. In turn, two reports based on nrITS and four low-copy nuclear loci place the *Contortae* either nested within subsection *Australes* with moderately high support (77–82% bootstrap support in Liston et al. [51]) or forming a polytomy with monophyletic subsections *Ponderosae * and *Australes *[53], respectively, while restriction fragment analysis including chloroplast, mitochondrial and nuclear DNA suggest a more derived position of subsection *Contortae* within section *Trifoliae * and some affinity to members of subsection *Australes*[50].

While our results cannot be considered conclusive by themselves, they certainly add important perspectives to *Pinus * evolutionary history as well as the use of plastome-scale sequences in plant phylogenomic analyses. For the genus *Pinus * as a whole, our dataset apparently represents the maximal resolution to be gained from the plastome, although various permutations of chloroplast loci may still prove useful at different levels of phylogenetic inquiry [for example see [98] and certainly plastome variation within-species warrants further interrogation for a number of species (i.e., *P*. *ponderosa P*. *lambertiana*, although see [98,103]). From this point, the next target of phylogenetic interrogation will likely be larger unique portions of the nuclear genome, particularly as increases in sequence output continue to outpace increases in read length for next-generation sequencers [104] and progress is made on the sequencing and assembly of a representative pine nuclear genome [105]. For the three specific clades investigated in this study, the patterns in response to variable site and long-branch removal from the full plastome alignment are intriguing and a measure of insight has been gained into the evolutionary histories and relationships of their plastomes, if not of the species themselves. In each case, decreasing the impact of phylogenetic noise by removing highly variable sites resulted in phylogenetic resolution more reflective of results based on nuclear and/or morphological data. At the same time, the impact of long-branch exclusion was less pronounced, suggesting that long-branch attraction artefacts are not prevalent at these levels of the *Pinus* phylogeny. The congruent results between model-based and parsimony methods for these clades also lend support to this conclusion, as methodological incongruence is another indication of possible long branch attraction artefacts [16]. This result is somewhat counter-intuitive, as all three lineages investigated have relatively long branches in chloroplast-based phylogenetic reconstruction (Additional File 4) [31]. It is possible that these long branches are not all reflective of the same biological processes. The long branches of *P*. *krempfii * and the *P*. *merkusii */*P*. *latteri * clade likely are due to relatively long periods of neutral divergence from their sister lineages. In these cases, however, it appears that removal of highly variable sites unmasks the limited underlying signal more definitively supporting their plastid-based resolutions - *P*. *krempfii* as sister to subsection *Gerardianae * of section *Quinquefoliae *, and *P*. *merkusii*/*P*. *latteri* as sister to subsection *Pinaster* of section *Pinus *. For subsection *Contortae*, on the other hand, chloroplast-based support for an early divergence in section *Trifoliae* is clearly inflated by the phylogenetic noise of highly variable sites. In this case, the pronounced effect of variable site removal combined with the relatively long branch leading to subsection *Contortae* may instead be indicative of elevated rates of evolution or responses to selection in this lineage, and a position sister to or within subsection *Australes * could be the final resolution of this challenging group.

## Conclusions

The promise of phylogenomics is still very much palpable and (to paraphrase Mark Twain) reports of its ‘demise’ [17,18] are greatly exaggerated. As demonstrated in the current study, a full-plastome matrix provides greatly increased resolution into the evolutionary history of the genus *Pinus *. Still, in this and other cases it is equally premature to confirm phylogenetic results based on genome-scale datasets without investigating first for the presence of misleading signal [17-19,22]. This is particularly important when trying to reconcile poorly supported topologies or conflicting phylogenetic results based on different sources or types of data [22], such as those represented by three historically recalcitrant taxa in the *Pinus* phylogeny. The present analysis and similar efforts [for example [35] also demonstrate not only the power of large (but well-managed) datasets to increase phylogenetic resolution, but the risk of relying on single sources of data, as inconsistencies between organellar- and nuclear-based analyses can remain even with greatly increased sampling. Fortunately, sequencing capacity and read length of next-generation platforms continue to increase [106-110], and combined with increasingly effective methods of genome interrogation [111-114] will make it easier to capture useful sequence data from what are currently less tractable genomes (such as plant nuclear and mitochondrial genomes). However, the development of analytical strategies to interrogate misleading signal present in large datasets will remain essential, as phylogenetic signal clearly is not always sufficient to overcome phylogenetic noise in identifying the relationships of certain taxa or organelles, even at genomic scales.

## Competing interests

Richard Cronn and Matthew Parks were invited speakers at Illumina User Group Meetings in 2009 and 2010, respectively, and received airfare, accommodation and meals for these events. The authors declare that they have no other competing interests, including funding, stocks/shares, patents or non-financial interests.

## Authors’ contributions

All authors conceived and developed the research plan. RC designed the hybridization methods, and MP and RC carried out template preparation and hybridization reactions. MP carried out post-sequencing assembly and analyses with contributions from AL. MP drafted the manuscript. All authors read and approved the final manuscript.

## Funding

This work was supported by the National Science Foundation grant number NSF ATOL-0629508.

## Supplementary Material

Additional file 1 Collection and sequencing information for accessions used in this study. Click here for file

Additional file 2 Settings used in AIR-Identifier. Click here for file

Additional file 3 ** Average per site rate variability of plastome regions for full plastome alignment using tree-dependent methodology.** Average per site category ranking for protein-coding exons, introns, rRNA and tRNA genes, and noncoding regions for full plastome alignment of 113 *Pinus* and Pinaceae species using tree-dependent methodology. Standard deviations are given in parentheses. Mean values with different superscript letters are significantly different at α < 0.05 in Tukey’s HSD test, following one-way ANOVA supporting different means at p < 0.0001. Click here for file

Additional file 4 ** Phylogenetic relationships within genus *Pinus* as determined from full plastome alignment.** a) Cladogram based on ML topology, showing support values below branches as ML bootstrap support / Bayesian posterior probability / parsimony bootstrap support. Support values are shown only for nodes with less than 100% bootstrap support and/or posterior probabilities less than 1.0; single values indicate either ML bootstrap support or Bayesian posterior probability. * indicates branch not supported in Bayesian or parsimony analysis. b) ML Phylogram with branch lengths determined from ML analysis; scale corresponds to probability of change per positions. Click here for file

Additional file 5 ** Trends in bootstrap support values and topologies for likelihood analyses of alignment partitions (tree-dependent).** For tree-dependent site variability analyses, the following are shown: a) Distributions of bootstrap support values for all nodes. Circles represent median bootstrap support for each A_n_ partition size. b) Distribution of branch score metric (triangles) and partition metric (circles) values for tests of topological congruence between A_n_ and corresponding B_n_ data partitions. Partition metric values shown are 0.1× actual value in order to fit on same scale with branch score values. Click here for file

Additional file 6 ** Distribution of bootstrap support for phylogenetic position of three clades in genus *Pinus * (tree-dependent).** Results for tree-dependent site variability analyses are shown for: a) subsection *Krempfianae *, b) *Pinus merkusii * / *P*. *latteri * and c) Subsection *Contortae *. In a), *P*. *krempfii * was found sister to subsection *Gerardianae* for all A_n_ partitions. In b), circles correspond to placement of *P*. *merkusii */*P*. *latteri * as sister to subsection *Pinaster * and triangles as sister to subsection *Pinus*. In c), circles correspond to placement of subsection *Contortae * as sister to subsection *Australes* and triangles as basal to both subsections *Australes * and *Contortae*; squares represent variable phylogenetic placements not including those represented by circles or triangles. For b) and c), monophyly of *P*. *merkusii*/*P*. *latteri* and subsection *Contortae* was supported at 100% bootstrap support for all A_n_ partitions. Click here for file

Additional file 7 ** Trends in corrected vs. uncorrected pairwise distances.** All pairwise distance values calculated as uncorrected pairwise distance and with a Jukes-Cantor correction, plotted for: a) all accessions used in study, b) genus *Pinus * accessions only, c) subgenus *Pinus * accessions only, d) subgenus *Strobus* accessions only, e) subsection *Quinquefoliae* and *Pinus monophylla * only, f) subsection *Pinus* and *Pinus ponderosa * only, and g) subsection *Trifoliae* and *Pinus thunbergii * only. Click here for file
