# Supplementary material for: Separating the wheat from the chaff: mitigating the effects of noise in a plastome phylogenomic data set from Pinus L. (Pinaceae)
Source: BMC Evol Biol. 2012 Jun 25;12:100. doi: 10.1186/1471-2148-12-100 (PMC3475122; doi:10.1186/1471-2148-12-100)
Supplement: Additional file 2 — Settings used in AIR-Identifier. [file 1471-2148-12-100-S2.doc]

**Additional File 2. Settings used in AIR-Identifier.**

**Parameter Setting**

outfile mlc

noisy 9

verbose 1

runmode 0

model 4 (HKY85)

Mgene 0

clock 0

fix_kappa 0

kappa 5

fix_alpha 0

alpha 0.5

Malpha 0

ncatG 8

fix_rho 0

rho 0.

nparK 0

nhomo 0

getSE 0

RateAncestor 1

Small_Diff 7e-6

cleandata 0

fix_blength 0

method 0
