# Supplementary material for: Separating the wheat from the chaff: mitigating the effects of noise in a plastome phylogenomic data set from Pinus L. (Pinaceae)
Source: BMC Evol Biol. 2012 Jun 25;12:100. doi: 10.1186/1471-2148-12-100 (PMC3475122; doi:10.1186/1471-2148-12-100)
Supplement: Additional file 3 — Average per site rate variability of plastome regions for full plastome alignment using tree-dependent methodology. Average per site category ranking for protein-coding exons, introns, rRNA and tRNA genes, and noncoding regions for full plastome alignment of 113 Pinus and Pinaceae species using tree-dependent methodology. Standard deviations are given in parentheses. Mean values with different superscript letters are significantly different at α < 0.05 in Tukey’s HSD test, following one-way ANOVA supporting different means at p < 0.0001. [file 1471-2148-12-100-S3.doc]

**Additional File 3.** **Average per site rate variability of plastome regions for full plastome alignment using tree-dependent methodology.**

|  | Noncoding regions | Protein-coding exons | Introns | tRNA | rRNA |
| --- | --- | --- | --- | --- | --- |
| average OV | 0 .19992^a^ (0.51810) | 0.06749^b^  (0.25397) | 0.10159^e^  (0.33825) | 0.06583^b, c^  (0.19460) | 0.02822^f^  (0.10961) |
| average OV without *ycf*1  without *ycf*1 or *ycf*2 |  | 0.05864^c^  (0.23104)  0 .04775^d^  (0.17196) |  |  |  |

Values given are for alignment of all 113 Pinus and Pinaceae accessions. Standard deviations are given in parentheses. Mean values with different superscript letters are significantly different at α<0.05 in Tukey’s HSD test, following one-way ANOVA supporting different means at p< 0.0001.
