# Supplementary figures and images for: Separating the wheat from the chaff: mitigating the effects of noise in a plastome phylogenomic data set from Pinus L. (Pinaceae)
Source: BMC Evol Biol. 2012 Jun 25;12:100. doi: 10.1186/1471-2148-12-100 (PMC3475122; doi:10.1186/1471-2148-12-100)

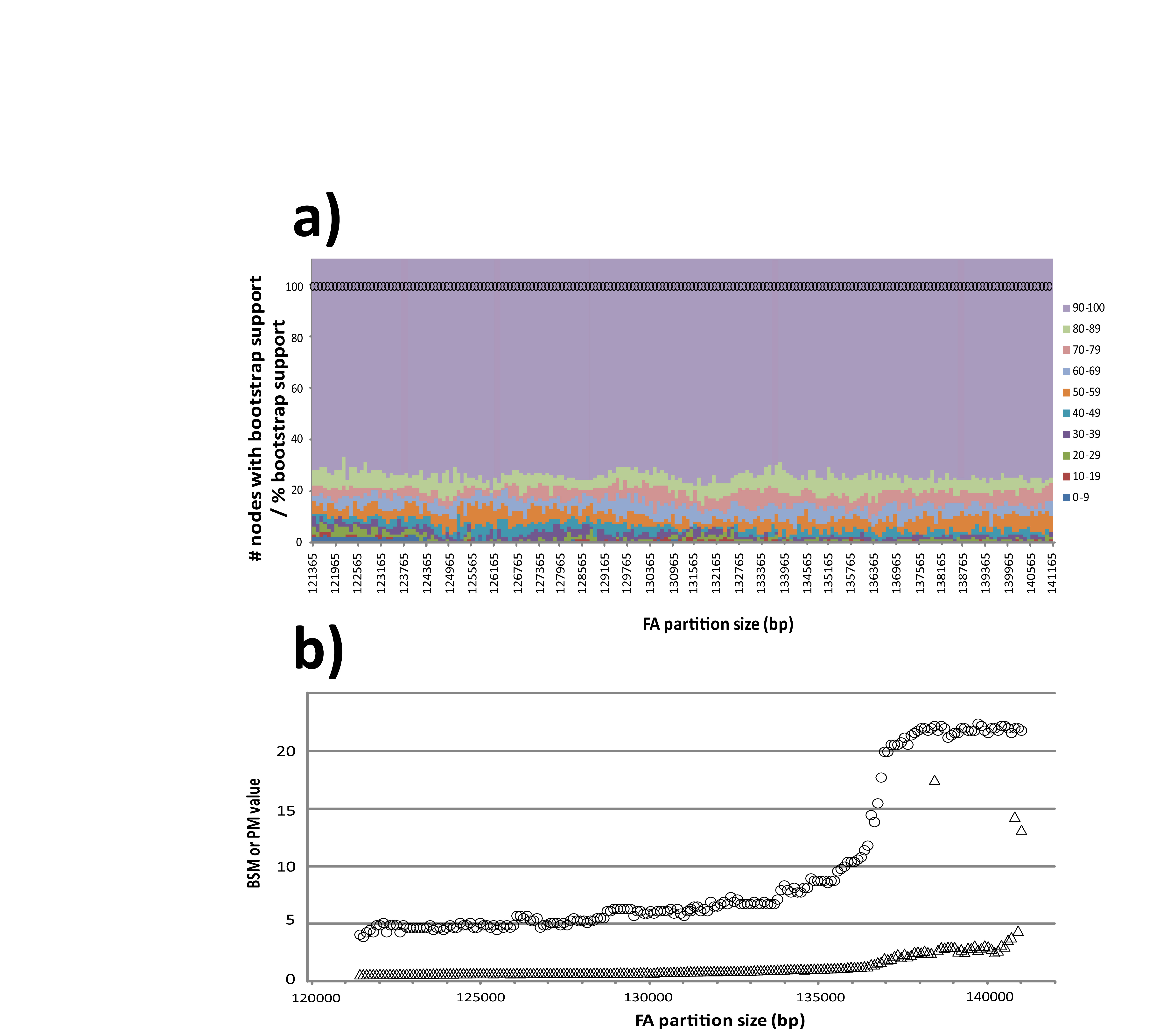

Supplement: Additional file 4 — Phylogenetic relationships within genus Pinus as determined from full plastome alignment. a) Cladogram based on ML topology, showing support values below branches as ML bootstrap support / Bayesian posterior probability / parsimony bootstrap support. Support values are shown only for nodes with less than 100% bootstrap support and/or posterior probabilities less than 1.0; single values indicate either ML bootstrap support or Bayesian posterior probability. * indicates branch not supported in Bayesian or parsimony analysis. b) ML Phylogram with branch lengths determined from ML analysis; scale corresponds to probability of change per positions. [file 1471-2148-12-100-S4.jpeg]

a)

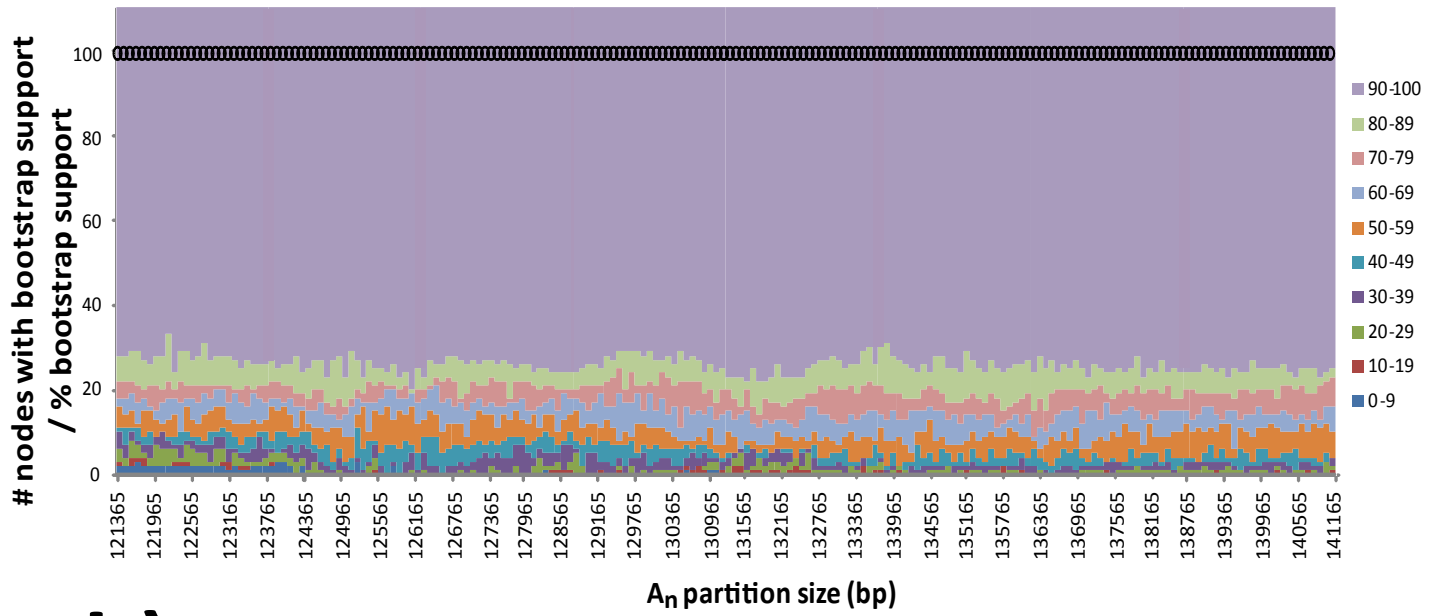

b)

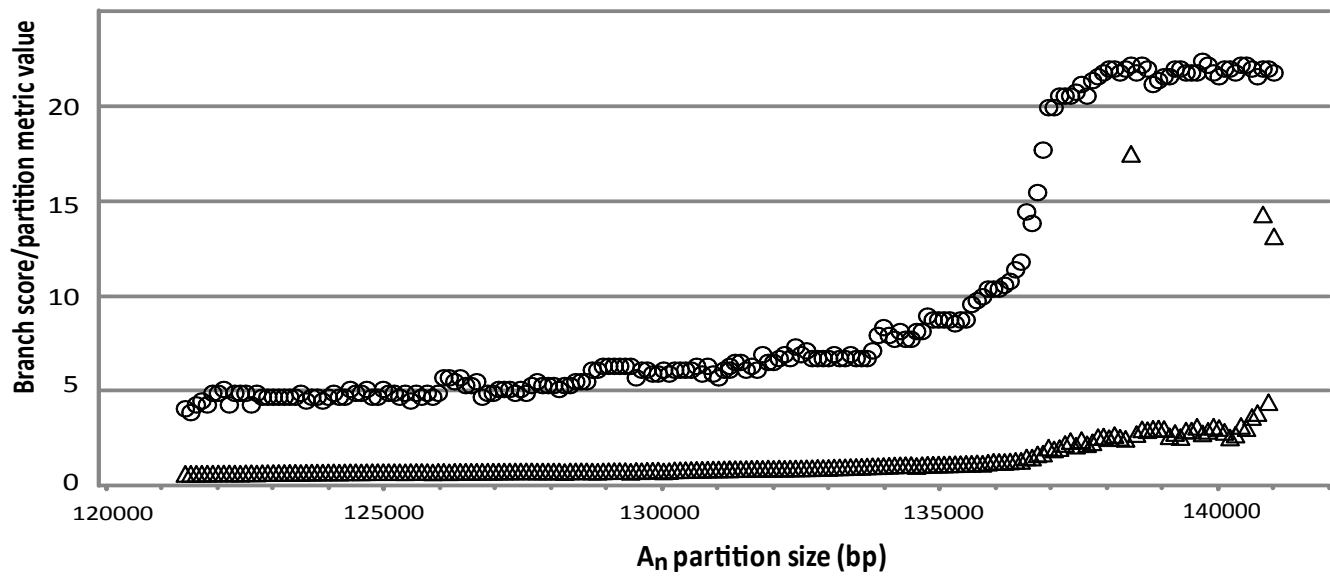

Supplement: Additional file 5 — Trends in bootstrap support values and topologies for likelihood analyses of alignment partitions (tree-dependent). For tree-dependent site variability analyses, the following are shown: a) Distributions of bootstrap support values for all nodes. Circles represent median bootstrap support for each An partition size. b) Distribution of branch score metric (triangles) and partition metric (circles) values for tests of topological congruence between An and corresponding Bn data partitions. Partition metric values shown are 0.1× actual value in order to fit on same scale with branch score values. [file 1471-2148-12-100-S5.pdf]

**a)**

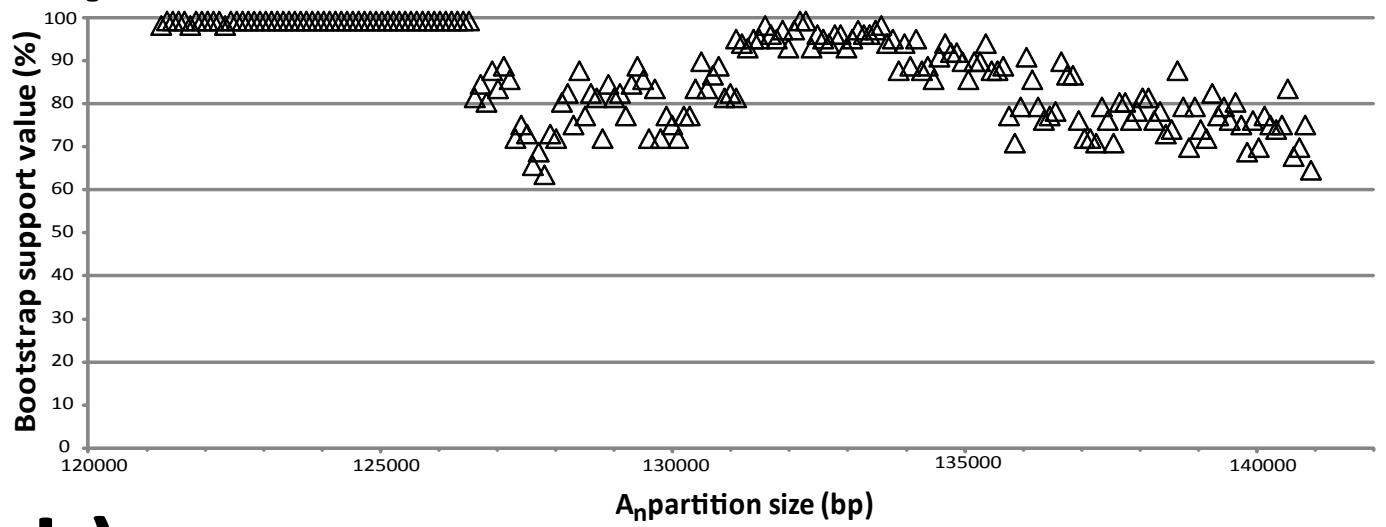

**b)**

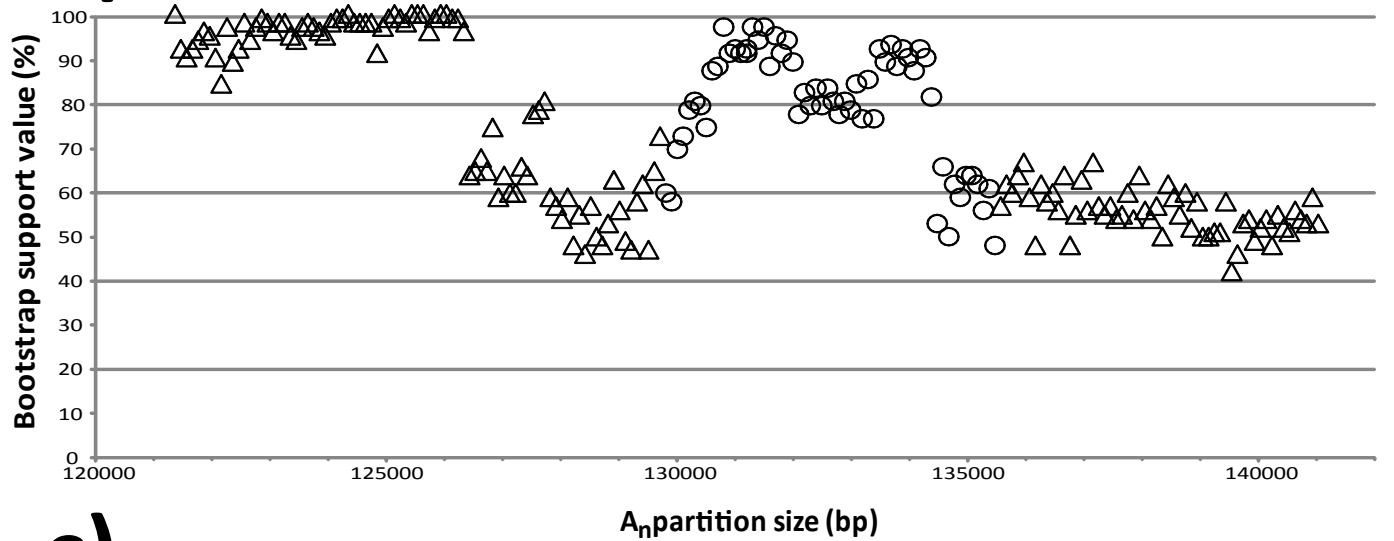

**c)**

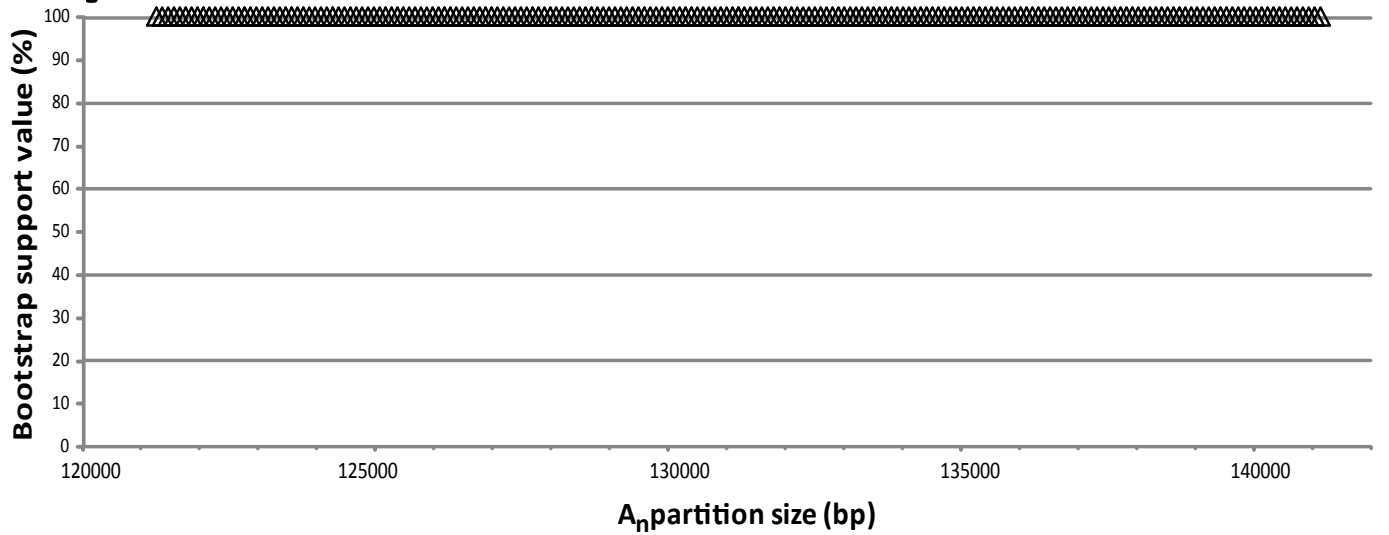

Supplement: Additional file 6 — Distribution of bootstrap support for phylogenetic position of three clades in genus Pinus (tree-dependent). Results for tree-dependent site variability analyses are shown for: a) subsection Krempfianae , b) Pinus merkusii / P. latteri and c) Subsection Contortae . In a), P. krempfii was found sister to subsection Gerardianae for all An partitions. In b), circles correspond to placement of P. merkusii /P. latteri as sister to subsection Pinaster and triangles as sister to subsection Pinus. In c), circles correspond to placement of subsection Contortae as sister to subsection Australes and triangles as basal to both subsections Australes and Contortae; squares represent variable phylogenetic placements not including those represented by circles or triangles. For b) and c), monophyly of P. merkusii/P. latteri and subsection Contortae was supported at 100% bootstrap support for all An partitions. [file 1471-2148-12-100-S6.pdf]
