# Supplementary material for: Separating the wheat from the chaff: mitigating the effects of noise in a plastome phylogenomic data set from Pinus L. (Pinaceae)
Source: BMC Evol Biol. 2012 Jun 25;12:100. doi: 10.1186/1471-2148-12-100 (PMC3475122; doi:10.1186/1471-2148-12-100)
Supplement: Additional file 7 — Trends in corrected vs. uncorrected pairwise distances. All pairwise distance values calculated as uncorrected pairwise distance and with a Jukes-Cantor correction, plotted for: a) all accessions used in study, b) genus Pinus accessions only, c) subgenus Pinus accessions only, d) subgenus Strobus accessions only, e) subsection Quinquefoliae and Pinus monophylla only, f) subsection Pinus and Pinus ponderosa only, and g) subsection Trifoliae and Pinus thunbergii only. [file 1471-2148-12-100-S7.doc]

**Additional File 7. Settings used in AIR-Identifier.**

**Parameter Setting**

outfile mlc

noisy 9

verbose 1

runmode 0

model 4 (HKY85)

Mgene 0

clock 0

fix_kappa 0

kappa 5

fix_alpha 0

alpha 0.5

Malpha 0

ncatG 8

fix_rho 0

rho 0.

nparK 0

nhomo 0

getSE 0

RateAncestor 1

Small_Diff 7e-6

cleandata 0

fix_blength 0

method 0
